# Impact of Healthcare Expenditures on Healthcare Outcomes in the Middle East and North Africa (MENA) Region: A Cross-Country Comparison, 1995–2015

**DOI:** 10.3389/fpubh.2020.624962

**Published:** 2021-02-04

**Authors:** Bander Balkhi, Dhfer Alshayban, Nawaf M. Alotaibi

**Affiliations:** ^1^Department of Clinical Pharmacy, College of Pharmacy, King Saud University, Riyadh, Saudi Arabia; ^2^Pharmacy Practice Department, College of Clinical Pharmacy, Imam Abdulrahman Bin Faisal University, Dammam, Saudi Arabia; ^3^Department of Pharmaceutics, Faculty of Pharmacy, Northern Border University, Rafha, Saudi Arabia

**Keywords:** healthcare resources, healthcare expenditures trend, Middle East-North Africa, life expectancy, cross-nation comparison

## Abstract

The association between healthcare expenditures and outcomes, mainly mortality and life expectancy, is complex. The real explanation for this association is not clear, especially in the Middle East and North Africa (MENA) region. This study assesses the impact of health expenditures on improving healthcare systems and health status and finds a relationship between health expenditures and health outcomes across different region. Annual time series data on healthcare spending and outcomes from 1995 to 2015 were used for MENA region in comparison to developed and developing countries. Health expenditure was adjusted by the consumer price index equation to the 2015 US dollar eliminate the impact of inflation on our results. For many countries, spending on healthcare continues to rise, Among MENA countries, we found that the United Arab Emirates and Kuwait spent more per capita on health, $1,711 and $1,420, respectively, than any other countries in the region. Although this study demonstrated a relationship between total healthcare expenditure and outcomes, some countries spend more on healthcare but have shorter life expectancy. In most countries, efficient and effective utilization of healthcare resources is the key strategy for improving health outcomes in any country. The lack of a positive correlation between healthcare spending and life expectancy may indicate that health resources are not allocated effectively. In those cases, increasing health spending does not guarantee that there is any kind of improvement in healthcare.

## Introduction

Over the last decades, healthcare expenditure has been increasing in many countries, and Middle East and North Africa (MENA) region follow the same pattern mainly because of the high price of health technology, increased awareness about health, and lifestyle changes. As a result, healthcare systems in MENA region are facing a huge challenge to keep up with the increasing demand for healthcare as results of rapid population growth, steady increase in elderly population and increase prevalence of chronic, non-communicable diseases (1). Population health can be affected by many elements, such as income, social and physiological factors, epidemiological factors, and accessibility to healthcare services. Healthcare spending as a factor that may have an impact on individual health is essential for effective policy making at the national and regional levels and for the sustainability of health services (2, 3).

There is no doubt that for any country, the healthcare system can play a significant role in the development process. Therefore, the increase in human capital stock investment is considered a vital factor for attaining the anticipated economic development in all countries (3, 4). It is a fact that healthier people can positively contribute to the growth of the economy because they are more productive and can live longer (4). As a result of the increasing life expectancy of the population, other health indicators such as infant and adult mortality rate will be improved.

Therefore, many countries invest a large percentage of their gross domestic product (GDP) on the health of their nations, as such investment could impact economic growth. The World Health Organization (WHO) has demonstrated the impact of improving overall patient health on economic growth. The WHO stated that for every 10% increase in life expectancy at birth, there was a 0.35% increase in economic growth per year. By contrast, sick people might have a negative impact on the economy, which could lead to a decline of about 50% in the growth rate between developed and developing countries (4).

The continued increase in health expenditure has resulted in a need to understand the impact on individual health outcomes as well as the need to know how limited resources can be used most efficiently (5). It is believed that better health outcomes for the population might indicate that a country has a more effective healthcare system, while a less healthy population might indicate that the country has an ineffective healthcare system (2). In other words, health expenditures are always thought to be in a direct relationship with healthcare outcomes, and several studies have assured this relationship; however, in some countries, other factors play important roles along with healthcare expenditures. Efficient resource utilization, environmental factors, education, and income are almost equally important as spending.

Understanding total healthcare expenditures is an important element for effective health care policy-making and strategy planning.

The impact of total health expenditure on the national economy has been addressed in several studies that examine the association between healthcare expenditure and health outcomes in developed and developing countries (6, 7).

The relationship between health expenditure and health outcome is complex and research studies showed controversial results on this association. Thus, the association between healthcare expenditures affect health outcomes is still not clear, especially in the MENA region which faced a major healthcare reform (8, 9). Comparing different countries around the world will be crucial in understanding this association. This study aimed to investigate the impact of healthcare expenditure on several health indicators across different countries, such as improving the healthcare system and overall health status, and to assess the relationship between health spending and health outcomes. Such studies will help policy makers in those countries assess whether they are spending too much on health in relation to health outcomes and promote the efficient use of health resources.

## Methods

### Study Design

A time series analysis was conducted to assesses the relationship between health expenditures and life expectancy at birth estimated by a cross-country comparison for the period−1995–2015 in MENA countries.

## Cross-Nation Comparison

### Data Sources and Data Analysis

The data were collected for a sample of developed and developing countries over 20 years (1995–2015). The countries were grouped according to the geographic region and per capita income.

#### Health Expenditure

Annual data on health expenditures and life expectancy at birth were extracted from two main databases: the World Bank's World Development Indicators and the WHO Health Statistics. The input for this study was the country's total healthcare expenditure, which is expressed as healthcare expenditures per capita (current US$). Health expenditure was adjusted by the consumer price index (CPI) equation to the 2015 US dollar to eliminate inflation.

#### Health Outcome

Mortality data is often used to assess population's health. The most commonly measure used to assessed mortality is the life expectancy in which it measures the expected year the person can live based considering person's specific characteristics. In this study, we defined health outcomes as life expectancy at birth. The average years of male and female population was used as an indicator of health status in each country. All required data were obtained from the WHO Health Statistics and World-Bank database. The study analyzed the relationship between healthcare expenditures per capita and health-outcomes. Data analysis was performed to assess the effect of health expenditure on life expectancy at birth.

In this study countries were classified according to their geographic location into seven areas; Europe, North America, Australia, South America, Middle East and North Africa (MENA), Africa and Asia. Our focus was on the Middle East and North Africa (MENA) region in comparison with other countries. In this study MENA countries includes the following countries: Algeria, Bahrain, Egypt, Iraq, Jordan, Kuwait, Lebanon, Libya, Morocco, Oman, Qatar, Saudi Arabia (SA), Syria, Tunisia, United Arab Emirates (UAE), and Yemen.

### Statistical Analysis

Data were analyzed using the Statistical Package for Social Studies (SPSS 22; IBM Corp., New York, NY, USA). Continuous variables were expressed as mean ± standard deviation. The *t*-test was used for continuous variables. Pearson correlation coefficients were used to assess the relationship between life expectancy and health expenditures for the period−1995–2015. A *p* < 0.05 was considered statistically significant.

### Ethical Review

Ethics approval was not required for this study as the study mainly used information freely available to public and do not use patient level data.

### Results

**Figure 1:**
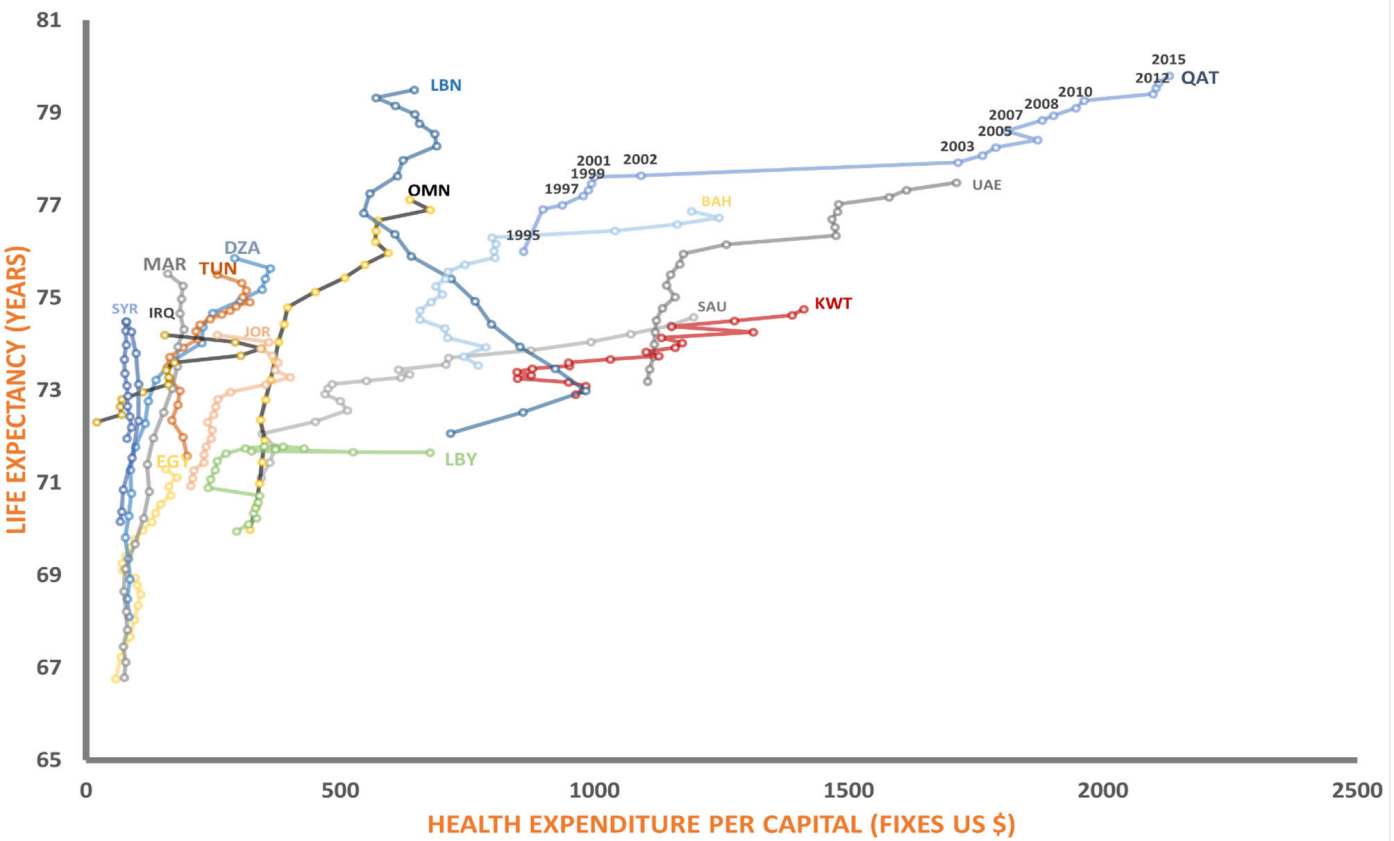
Relationship between health expenditure and life expectancy. SAU, Saudi Arabia; OMN, Oman; UAE, United Arab Emirates; KWT, Kuwait; LBN, Lebanon; JPR, Jordan; IRQ, Iraq; QAT, Qatar; SYR, Syrian; BHR, Bahrain; YEM, Yemen; EGY, Egypt; LBY, Libya; DZA, Algeria; TUN, Tunisia; MAR, Morocco.

Over the study period, we observed an overall improvement in life expectancy at birth in majority of the countries included in this study. Figure 1 shows the association between per capita health expenditures and life expectancies for MENA countries at different points in time. Overall, the health expenditure and life expectancy has been improved in the MENA region since 1995.

Over the study period (1995–2015), life expectancy increased by an average of 5 years, with a range of 1 year in Iraq to 9 years in Morocco. The average life expectancy at birth in the MENA region in 2015 was 74.8 years, ranging from 64.2 years in Sudan to 79.5 years in Lebanon.

The health care expenditure also on the rise and Qatar, United Arab Emirates, spend more per capita on health $2,029 and $1,420, respectively, than any other country in the region. While Syria and Yemen have the lowest spending per capita on health in the region. From Figure 1 it is been observed that despite the large variabilities, we noticed that higher expenditure of health not always associated with increase in health outcome, as, some countries spend more on healthcare but have shorter life expectancy and other were spend less on health while they have longer life expectancy.

In order to compare MENA region with other countries, we divided the countries into seven areas, and the total distribution of healthcare expenditure was presented as the mean and standard deviation (SD) for the whole study period (Table 1). Our finding indicated that spending on healthcare continued to rise in all regions, the highest per-capita healthcare spending was in the Europe and Australia, with the highest healthcare spending ranging from $4,717 to $4,412 per capita. While the lowest per-capita healthcare spending was observed in Africa region $128.3 per capita. The life expectancy was highest in Europe and Australia 80.2 and 80.5 years in 2015 and only 67.2 years in Africa in 2015 (Table 2).

**Table 1:** Mean and standard deviation of health care expenditure for a period of 1995–2015 in the seven areas.

**Year**	**Europe**		**North America**			**Australia**			**South America**			**MENA**			**Africa**			**Asia**		
	**Mean**	**SD**	**Mean**	**SD**	***P*-value^1^**	**Mean**	**SD**	***P*-value^2^**	**Mean**	**SD**	***P*-value^3^**	**Mean**	**SD**	***P*-value^4^**	**Mean**	**SD**	***P*-value^5^**	**Mean**	**SD**	***P*-value^6^**
1995	3641.5	1488.4	2295.0	2698.4	0.2	2474.5	0.0	0.5	374.2	162.0	0.014^*^	520.3	395.1	<0.001^*^	87.6	89.9	<0.001^*^	608.0	1448.2	<0.001^*^
1996	3629.6	1392.0	2296.6	2721.5	0.2	2702.8	0.0	0.5	418.9	147.3	0.011^*^	540.0	405.4	<0.001^*^	93.2	93.8	<0.001^*^	542.0	1212.2	<0.001^*^
1997	3216.3	1108.1	2341.9	2761.3	0.4	2639.7	0.0	0.6	443.4	110.8	0.007^*^	554.4	417.8	<0.001^*^	92.9	96.4	<0.001^*^	511.6	1122.8	<0.001^*^
1998	3253.9	1115.7	2371.2	2833.1	0.4	2345.4	0.0	0.5	401.7	117.6	0.006^*^	538.4	402.7	<0.001^*^	95.9	95.6	<0.001^*^	445.6	1040.5	<0.001^*^
1999	3240.1	1087.7	2450.8	2898.6	0.5	2525.6	0.0	0.5	304.6	52.7	0.004^*^	531.7	371.1	<0.001^*^	89.5	100.4	<0.001^*^	512.2	1209.4	<0.001^*^
2000	2888.3	938.5	2538.1	2958.8	0.7	2402.9	0.0	0.6	281.3	113.6	0.004^*^	514.7	348.0	<0.001^*^	87.2	101.0	<0.001^*^	546.8	1278.7	<0.001^*^
2001	2897.2	962.9	2625.1	3078.0	0.8	2228.4	0.0	0.5	246.6	78.5	0.004^*^	521.0	364.1	<0.001^*^	87.7	100.8	<0.001^*^	494.6	1107.9	<0.001^*^
2002	3181.9	1055.2	2773.8	3285.9	0.7	2481.1	0.0	0.5	220.5	62.9	0.003^*^	542.1	390.5	<0.001^*^	79.8	74.3	<0.001^*^	488.8	1056.0	<0.001^*^
2003	3882.4	1177.4	2994.8	3469.5	0.5	3053.8	0.0	0.5	221.7	71.4	0.002^*^	623.6	543.0	<0.001^*^	87.7	76.8	<0.001^*^	535.3	1136.8	<0.001^*^
2004	4337.6	1237.5	3164.1	3603.9	0.4	3680.1	0.0	0.6	251.9	95.0	0.001^*^	634.8	554.7	<0.001^*^	96.9	82.1	<0.001^*^	575.8	1202.8	<0.001^*^
2005	4364.0	1185.1	3356.1	3659.8	0.4	3900.3	0.0	0.7	356.9	166.8	0.001^*^	650.3	550.0	<0.001^*^	100.4	80.2	<0.001^*^	597.6	1172.3	<0.001^*^
2006	4436.9	1129.0	3512.1	3778.8	0.5	4022.8	0.0	0.7	418.6	215.1	0.001^*^	670.8	539.0	<0.001^*^	110.4	83.1	<0.001^*^	606.0	1099.8	<0.001^*^
2007	4872.5	1182.7	3729.0	3834.3	0.4	4662.1	0.0	0.9	509.8	248.4	0.001^*^	697.2	518.2	<0.001^*^	130.3	91.4	<0.001^*^	635.7	1089.9	<0.001^*^
2008	5214.4	1342.7	3758.2	3846.4	0.3	4855.0	0.0	0.8	592.5	277.9	0.001^*^	737.6	524.5	<0.001^*^	149.6	103.8	<0.001^*^	687.2	1182.7	<0.001^*^
2009	5111.2	1472.7	3806.8	3962.2	0.4	4703.1	0.0	0.8	603.8	289.8	0.002^*^	793.9	577.5	<0.001^*^	155.6	115.1	<0.001^*^	738.8	1348.4	<0.001^*^
2010	5019.9	1589.4	4018.5	4112.5	0.5	5785.9	0.0	0.7	729.2	382.1	0.004^*^	742.6	497.1	<0.001^*^	167.0	124.4	<0.001^*^	826.6	1465.5	<0.001^*^
2011	5481.4	1998.9	4070.3	4120.7	0.4	6711.1	0.0	0.6	808.7	428.8	0.010^*^	790.1	564.7	<0.001^*^	166.9	113.3	<0.001^*^	911.7	1603.7	<0.001^*^
2012	5136.4	1966.7	4050.7	4175.2	0.5	6754.6	0.0	0.5	790.5	319.9	0.013^*^	842.1	623.9	<0.001^*^	205.3	195.7	<0.001^*^	926.8	1603.3	<0.001^*^
2013	5302.3	2054.2	4053.6	4142.6	0.5	6367.1	0.0	0.6	784.8	319.5	0.014^*^	860.0	639.1	<0.001^*^	194.4	156.5	<0.001^*^	857.5	1346.2	<0.001^*^
2014	5272.0	2016.9	4051.5	4168.9	0.5	6037.8	0.0	0.7	759.2	267.8	0.012^*^	892.8	656.9	<0.001^*^	179.9	127.1	<0.001^*^	828.1	1266.6	<0.001^*^
2015	4717.4	2066.2	3644.3	4412.0	0.5	4535.8	0.0	0.9	577.3	287.2	0.021^*^	822.7	628.1	<0.001^*^	128.3	94.7	<0.001^*^	784.1	1271.6	<0.001^*^

*Europe was used as a reference when we calculated p-values for other areas*.

* *Significant p-value*.

1 *P value was calculated between Europe & North America*.

2 *P value was calculated between Europe & Australia*.

3 *P value was calculated between Europe & South America*.

4 *P value was calculated between Europe & Middle east*.

5 *P value was calculated between Europe & Africa*.

6 *P value was calculated between Europe & Asia*.

**Table 2:** Mean and standard deviation of life expectancy for a period of 1995–2015 in the seven areas.

**Year**	**Europe**		**North America**			**Australia**			**South America**			**MENA**			**Africa**			**Asia**		
	**Mean**	**SD**	**Mean**	**SD**	***P*-value^1^**	**Mean**	**SD**	***P*-value^2^**	**Mean**	**SD**	***P*-value^3^**	**Mean**	**SD**	***P*-value^4^**	**Mean**	**SD**	***P*-value^5^**	**Mean**	**SD**	***P*-value^6^**
1995	77.3	1.1	75.4	2.1	0.1	77.8	0.0	0.6	68.5	1.3	<0.001^*^	70.7	4.4	0.001^*^	60.3	9.6	<0.001^*^	67.3	6.3	0.001^*^
1996	77.6	1.1	75.8	2.1	0.1	78.1	0.0	0.7	68.9	1.2	<0.001^*^	71.0	4.4	<0.001^*^	60.5	9.7	<0.001^*^	67.8	6.3	0.002^*^
1997	77.8	1.1	76.1	2.1	0.1	78.5	0.0	0.6	69.3	1.0	<0.001^*^	71.3	4.3	<0.001^*^	60.7	9.8	<0.001^*^	68.2	6.3	0.002^*^
1998	78.0	1.1	76.3	2.0	0.1	78.6	0.0	0.6	69.7	0.9	<0.001^*^	71.6	4.3	<0.001^*^	60.9	9.9	<0.001^*^	68.6	6.2	0.002^*^
1999	78.2	1.1	76.5	2.0	0.1	78.9	0.0	0.6	70.1	0.8	<0.001^*^	71.9	4.3	0.001^*^	61.2	9.9	<0.001^*^	68.8	6.2	0.002^*^
2000	78.5	1.1	76.7	2.0	0.1	79.2	0.0	0.5	70.5	0.7	<0.001^*^	72.1	4.3	<0.001^*^	61.5	9.9	<0.001^*^	69.2	6.3	0.002^*^
2001	78.8	1.1	76.9	2.0	0.047^*^	79.6	0.0	0.5	70.9	0.6	<0.001^*^	72.4	4.2	<0.001^*^	61.8	9.9	<0.001^*^	69.5	6.4	0.002^*^
2002	78.9	1.1	77.1	2.0	0.1	79.9	0.0	0.4	71.2	0.5	<0.001^*^	72.7	4.2	0.001^*^	62.2	9.8	<0.001^*^	69.8	6.4	0.003^*^
2003	79.0	1.1	77.3	2.0	0.1	80.2	0.0	0.3	71.5	0.4	<0.001^*^	72.9	4.2	0.001^*^	62.7	9.7	<0.001^*^	70.2	6.5	0.003^*^
2004	79.5	1.2	77.6	2.1	0.045^*^	80.5	0.0	0.4	71.8	0.3	<0.001^*^	73.2	4.2	<0.001^*^	63.1	9.5	<0.001^*^	70.5	6.5	0.003^*^
2005	79.7	1.1	77.7	2.1	0.039^*^	80.8	0.0	0.3	72.1	0.2	<0.001^*^	73.4	4.1	<0.001^*^	63.6	9.4	<0.001^*^	70.8	6.5	0.003^*^
2006	80.0	1.1	77.9	2.0	0.025^*^	81.0	0.0	0.4	72.4	0.1	<0.001^*^	73.6	4.1	<0.001^*^	64.0	9.2	<0.001^*^	71.3	6.5	0.003^*^
2007	80.2	1.1	78.1	2.0	0.029^*^	81.3	0.0	0.4	72.7	0.0	<0.001^*^	73.8	4.1	<0.001^*^	64.5	9.1	<0.001^*^	71.6	6.4	0.004^*^
2008	80.4	1.1	78.3	2.0	0.026^*^	81.4	0.0	0.4	73.0	0.1	<0.001^*^	74.0	4.0	<0.001^*^	64.9	8.9	<0.001^*^	71.9	6.3	0.004^*^
2009	80.6	1.1	78.5	2.1	0.027^*^	81.5	0.0	0.4	73.3	0.2	<0.001^*^	74.1	4.0	<0.001^*^	65.3	8.7	<0.001^*^	72.2	6.3	0.004^*^
2010	80.9	1.1	78.7	2.1	0.023^*^	81.7	0.0	0.5	73.5	0.3	<0.001^*^	74.2	4.0	<0.001^*^	65.7	8.6	<0.001^*^	72.4	6.2	0.003^*^
2011	81.4	1.0	78.8	2.2	0.001^*^	81.9	0.0	0.6	73.8	0.5	<0.001^*^	74.3	4.1	<0.001^*^	66.0	8.5	<0.001^*^	72.7	6.1	0.003^*^
2012	81.4	1.0	79.0	2.1	0.011^*^	82.0	0.0	0.5	74.0	0.6	<0.001^*^	74.4	4.1	<0.001^*^	66.3	8.4	<0.001^*^	73.0	6.1	0.003^*^
2013	81.6	1.1	79.1	2.2	0.010^*^	82.1	0.0	0.6	74.3	0.6	<0.001^*^	74.5	4.2	<0.001^*^	66.6	8.3	<0.001^*^	73.3	6.1	0.003^*^
2014	82.0	1.0	79.2	2.2	0.005^*^	82.3	0.0	0.8	74.5	0.7	<0.001^*^	74.7	4.2	<0.001^*^	66.9	8.1	<0.001^*^	73.6	6.1	0.003^*^
2015	82.2	1.0	79.3	2.2	0.004^*^	82.5	0.0	0.8	74.7	0.7	<0.001^*^	74.8	4.2	<0.001^*^	67.2	8.0	<0.001^*^	73.8	6.1	0.003^*^

* *Significant p-value*.

1 *P value was calculated between Europe & North America*.

2 *P value was calculated between Europe & Australia*.

3 *P value was calculated between Europe & South America*.

4 *P value was calculated between Europe & Middle east*.

5 *P value was calculated between Europe & Africa*.

6 *P value was calculated between Europe & Asia*.

**Figure 2:**
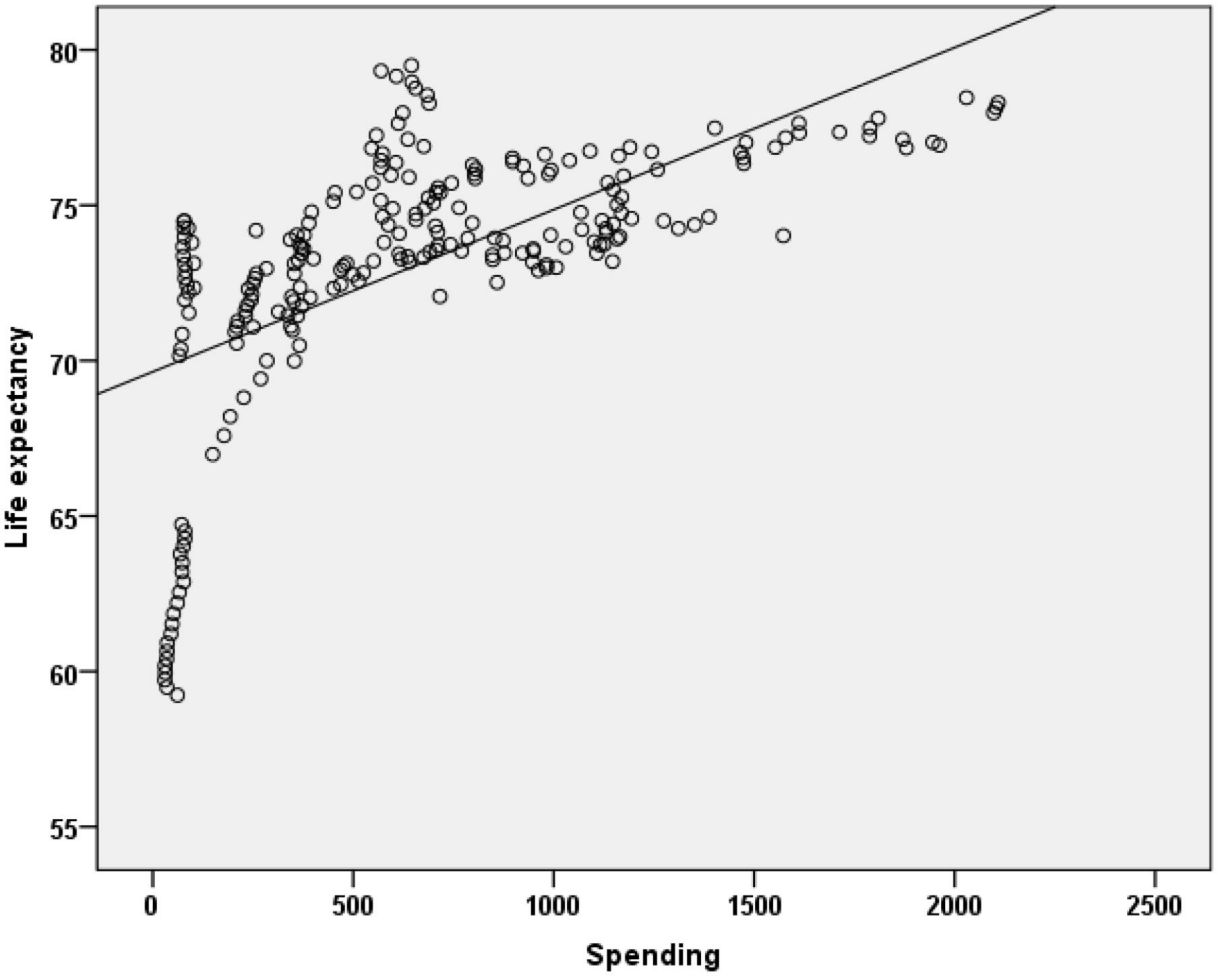
Relationship between life expectancy and health exenditures for a period of 1995–2015 in MENA Countries.

Pearson correlation coefficients were calculated between health spending and outcomes for each regions for the period 1995–2015 are presented in Table 3. The overall expenditure on healthcare was significantly correlated with life expectancy. Overall, country spending on health was positively correlated with life expectancy (*r* = 0.610, *p* < 0.001), while the correlation for MENA countries is a higher (*r* = 0.624, *p* < 0.001). This univariate correlation is shown in Figure 2 for all countries included in the study. The correlation was more significant for Australia and MENA countries compared to other regions included in this study.

**Table 3:** Relationship between life expectancy and health expenditures for a period of 1995–2015.

**Areas**	***r***	***P*-value**
World	0.610^**^	<0.001
Europe	0.396^**^	<0.001
North America	0.426^**^	<0.001
Australia	0.872^**^	<0.001
South America	0.555^**^	<0.001
MENA	624^**^	<0.001
Africa	0.671^**^	<0.001
Asia	0.769^**^	<0.001

** *Correlation is significant at the 0.01 level (2-tailed)*.

*r = Pearson Correlation coefficient*.

## Discussion

In this study, we examine the health expenditures of MENA countries and focusing on investigate the relationship between healthcare expenditures and health outcomes, which are measured as life expectancy here. We observed a large variation in health expenditure and health outcome across different countries.

Overall we noticed that for majority of the countries the total health expenditure was increased overtime and at the same time life expectancy tend to be improved over years. Our results demonstrated that among MENA countries Lebanon appears to hold the most successful position in terms of highest life expectancy at a reasonable level of healthcare expenditure. Qatar and UAE were outlier as they spend more and their life expectancy is similar to some MENA counties that spend less.

On the other hand, Europe demonstrate somewhat variable, but mostly comparable curves. The health expenditures have been increased in Europe over years from $3,641.5 in 1995 to $4717 per capita in 2015. Over the same period, the average life expectancy area has increased from 77.3 years to 82.8 years. The UK eventually reducing the per capita costs while maintaining the desired life expectancy level. Other countries appear to achieve the same level of life expectancy with additional investments. This results are in line with previous studies that find a positive relationship between increase spending on health and health outcome in Europe countries.

MENA countries spend less on healthcare compare to high income countries. According to the latest report of the world bank the average healthcare expenditure as a percentage of GDP in MENA countries is 5.96% of GDP which is lower than other regions North America 16.42%, OCED 12.46% and Europe 10.14% and East Asia and Pacific 6.67% as share of GDP. Within MENA region we notice a large variation of health care spending as percentage of GDP. Lebanon, Tunis and Saudi Arabia spend 8.35, 7.29, and 6.36% of GDP on health, respectively, which is relatively higher compared to other MENA countries UAE (3.3%), Kuwait (2.7%), Oman (2.3%), and Qatar (2.49). The lower healthcare spending in MENA region compared to others, could be an incentive for policy maker to improve healthcare expenditure in order to improve population health.

The two countries utilizing the greatest healthcare resources are Switzerland and the US, with a striking difference in life expectancy given the same healthcare investments.

The population in the US has demonstrated higher mortality and morbidity indices than other high-income countries. Excess death rates in the age groups younger than 40 years have been thought to largely contribute to the large variation in life expectancy between the US and other developed countries (10, 11).

US healthcare spending constituted 17.8% of the gross domestic product, whereas the figures were dramatically lower in Australia and Switzerland (9.6 and 12.4%, respectively).

US also ranked lower in overall health insurance and highest (55%) in private health insurance coverage. US healthcare costs per capita are almost twice as high as those of its European counterparts (12). The main reason for such different between MENA countries and US is due to the differences in work-force financing strategies, drugs and medical equipment being marketed at substantially higher prices in US, deliberate ordering of expensive medical investigations, and perhaps lack of governmental regulation. The comparative analysis by Papanicolas et al. (12) revealed that the salaries of healthcare providers were almost double in US compared to their European counterparts. Furthermore, the administrative costs (accounting for 8%) were significantly higher in US compared to a range of 1–3% in the peer countries. Another reason for high healthcare costs is that in the US, more subspecialty consults are ordered, which results in dramatically increased overall healthcare costs (12, 13).

During the study period we observed that the average life expectancy in MENA area has increased by about 5 years with Lebanon stands out with the highest achieved life expectancy at the lowest per capita expenses, as demonstrated by the almost vertical curve topping its contemporaries.

The slopes of the curves acquired from the other MENA countries (i.e., Saudi Arabia, Kuwait, and the United Arab Emirates) are more reminiscent of Japan and Germany, indicating a similar pace of development. This perhaps stems from the fact that, in contrast to the previously mentioned MENA counterparts, these represent developed countries, which are likely to follow the curves of their European peers given that the appropriate healthcare systems are most efficiently integrated.

Our results show a positive effect for health expenditure on the life expectancy for MENA region. This relationship were consistent with the previous study which indicated that a unit increase in per capita health expenditure in MENA countries reduces the mortality rate by almost 9.5 deaths per 1,000 live births (7, 14). Although, we observed an improvement in life expectancy in MENA countries, the progress in Iraq, Yemen and Syria is quite low. For countries with lower spending on health previous study indicated that even a small amounts of health expenditure, would have a bigger impact on health outcome (15). A study that pooled a data for 50 developing countries indicated a strong association between health expenditure and infant mortality rates (16). Another studies demonstrated that health expenditure has statistically significant effect impact on under-five mortality and maternal mortality. This should be taken into consideration by health policy maker when allocate health funding (17).

The role of the government's involvement in healthcare acquisition is a widely recognized factor in healthcare cost generation and can be exemplified by the Canadian and German healthcare systems. It appears that systems utilizing government provision and insurance achieve lower per capita costs and universal accessibility. By contrast, 40 million US citizens are deprived of health insurance and must pay out of pocket for treatment. Furthermore, Canada and Germany have demonstrated higher life expectancies and lower infant mortality rates compared to US (13).

It follows from the discussion above that the differences in healthcare system organization can affect the life expectancy / per capita healthcare expenditure ratio; therefore, this should be considered for MENA countries, especially for those with developing healthcare systems. As can be seen from Figure 1, in countries at the lowest end of per capita healthcare spending (Yemen, Egypt, Bahrain, and Lebanon) even small investments in healthcare can substantially boost the population life expectancy and catch up with modern standards (18). This stems from the substantial differences in the healthcare problems that these countries are currently facing. Minor improvements in overall living conditions as well as providing widely accessible primary preventive healthcare, can dramatically increase the overall survival rate and therefore the health expectancy at birth (19). This contrasts with cancer prevention and management of chronic conditions that require continuous specialized medical attention. In other words, the current healthcare systems in these countries appear to have an evolutionary disadvantage, but when modern medical knowledge and achievements are properly provided, these countries may quickly transition to a state similar to their counterparts in Europe.

A study that assessed inefficiency of health systems in MENA region demonstrated that most MENA countries appear to have reasonably high degree of technical efficiency with Morocco, Lebanon and Qatar, are in the top of the countries in term of technically efficiency. On the other hand, worst performers were observed in Yemen and Sudan. This indicate that there is still a room for healthcare system efficiency improvement in MENA region (20).

In general our results were in line with previous studies about the positive impact of health spending on health outcome.

Increasing in life expectancy on the other hand is associated with increasing in the utilization of health service by aging population who is the largest users of health services. In terms of life expectancy previous studies found that females gained on average 4–6-year over men on the OCED countries this may due to the fact many prevention health program were focusing on female like breast and cervical cancer screening and other educational program (15).

Other studies demonstrated a positive relationship between increase spending on pharmaceutical product have a positive impact on health outcomes (21, 22). This association required further investigation specially with many countries shift to value based health care and use both clinical and economic evaluation in medication reimbursement decision which can improve efficiency in health funding and resource allocation (23). Health Technology assessment is still in early phase in MENA region but it anticipates to play a major role in the near future in optimize healthcare resource allocation specially with the high cost of innovative health technology (24). The current health reforms program in many MENA countries would play an important role in improve health outcome while maintains health costs. Future research should examine the impact of such program and identified factors that contribute to the large variation of health outcomes in the region.

## Conclusion

A large variation was demonstrated between health expenditures per capita and life expectancy in MENA countries, and this variation is growing with time.

Overall our study demonstrated that health expenditure is an important factors in improving health outcome in MENA region. Efficient allocation of health resources is crucial for stability of any health care system and finding of this study have important policy implications for MENA countries. Policymakers are concerned with measuring the extent to which the increase in health expenditure is keeping pace with the increase in health outcome, which is an important factor for assessing if a country has an efficient health system and determining the gap to reach optimum levels for MENA countries with poor health status.

## Data Availability Statement

The original contributions presented in the study are included in the article/Supplementary Material, further inquiries can be directed to the corresponding author/s.

## Author Contributions

BB, DA, and NA design the study. DA and NA collect the data and edit and proof read the manuscript. BB and DA analyzed the data. BB wrote the article. All authors contributed to the article and approved the submitted version.

## Conflict of Interest

The authors declare that the research was conducted in the absence of any commercial or financial relationships that could be construed as a potential conflict of interest.
