# Supplementary material for: Impact of Healthcare Expenditures on Healthcare Outcomes in the Middle East and North Africa (MENA) Region: A Cross-Country Comparison, 1995–2015
Source: Front Public Health. 2021 Feb 4;8:624962. doi: 10.3389/fpubh.2020.624962 (PMC7890180; doi:10.3389/fpubh.2020.624962)
Supplement: Supplementary file 1 [file Data_Sheet_1.docx]

**Appendix A. health care expenditure in MENA from 1995-2015**

| **Country Name** | **Saudi Arabia** | **Oman** | **United Arab Emirates** | **Kuwait** | **Lebanon** | **Jordan** | **Iraq** | **Qatar** | **Syria** | **Bahrain** | **Yemen, Rep.** | **Egypt** | **Sudan** | **Libya** | **Algeria** | **Tunisia** | **Morocco** |
| --- | --- | --- | --- | --- | --- | --- | --- | --- | --- | --- | --- | --- | --- | --- | --- | --- | --- |
| **1995** | $ 344.13 | $ 322.00 | $ 1,104.00 | $ 961.86 | $ 716.16 | $ 205.21 | - | $ 935.74 | $ 79.49 | $ 770.05 | $ 61.67 | $ 57.92 | $ 32.24 | $ 295.61 | $ 83.78 | $ 197.90 | $ 75.01 |
| **1996** | $ 361.02 | $ 335.00 | $ 1,107.39 | $ 977.00 | $ 858.74 | $ 209.60 | - | $ 987.05 | $ 88.53 | $ 742.44 | $ 35.24 | $ 68.46 | $ 22.04 | $ 318.51 | $ 80.74 | $ 190.58 | $ 78.27 |
| **1997** | $ 372.02 | $ 341.00 | $ 1,115.61 | $ 981.93 | $ 982.03 | $ 211.14 | - | $ 993.15 | $ 85.54 | $ 785.65 | $ 30.00 | $ 85.75 | $ 24.47 | $ 334.68 | $ 85.56 | $ 167.74 | $ 73.23 |
| **1998** | $ 345.84 | $ 346.00 | $ 1,117.00 | $ 947.69 | $ 922.07 | $ 231.72 | - | $ 924.30 | $ 80.48 | $ 710.57 | $ 31.18 | $ 94.69 | $ 24.40 | $ 328.98 | $ 83.01 | $ 180.14 | $ 80.52 |
| **1999** | $ 450.67 | $ 351.00 | $ 1,119.00 | $ 847.91 | $ 852.58 | $ 231.28 | - | $ 897.45 | $ 81.48 | $ 704.99 | $ 30.60 | $ 102.42 | $ 21.25 | $ 332.73 | $ 77.23 | $ 184.38 | $ 78.68 |
| **2000** | $ 513.49 | $ 343.00 | $ 1,120.50 | $ 875.00 | $ 796.92 | $ 235.81 | - | $ 897.08 | $ 79.68 | $ 656.34 | $ 34.78 | $ 106.97 | $ 20.70 | $ 338.05 | $ 84.37 | $ 164.42 | $ 73.58 |
| **2001** | $ 499.57 | $ 353.15 | $ 1,133.00 | $ 846.89 | $ 764.17 | $ 245.23 | - | $ 977.67 | $ 76.15 | $ 656.07 | $ 35.62 | $ 100.31 | $ 21.63 | $ 340.72 | $ 89.00 | $ 159.65 | $ 76.60 |
| **2002** | $ 469.24 | $ 363.28 | $ 1,158.37 | $ 876.73 | $ 717.25 | $ 247.45 | - | $ 1,090.46 | $ 75.31 | $ 678.50 | $ 36.15 | $ 95.96 | $ 23.81 | $ 238.98 | $ 87.19 | $ 164.40 | $ 95.70 |
| **2003** | $ 475.51 | $ 370.69 | $ 1,141.00 | $ 949.98 | $ 639.28 | $ 238.42 | $ 21.13 | $ 1,879.17 | $ 78.66 | $ 700.46 | $ 45.10 | $ 71.47 | $ 28.78 | $ 244.82 | $ 97.17 | $ 190.62 | $ 113.05 |
| **2004** | $ 483.65 | $ 377.65 | $ 1,148.52 | $ 948.61 | $ 606.41 | $ 250.62 | $ 70.05 | $ 1,961.86 | $ 77.47 | $ 687.32 | $ 48.16 | $ 70.34 | $ 36.38 | $ 253.45 | $ 115.62 | $ 219.71 | $ 123.83 |
| **2005** | $ 550.69 | $ 389.26 | $ 1,167.00 | $ 1,030.03 | $ 545.53 | $ 254.44 | $ 67.33 | $ 1,945.63 | $ 77.40 | $ 705.84 | $ 51.62 | $ 77.43 | $ 42.39 | $ 257.20 | $ 121.78 | $ 215.81 | $ 120.24 |
| **2006** | $ 618.73 | $ 395.95 | $ 1,174.17 | $ 1,125.34 | $ 557.71 | $ 258.78 | $ 69.75 | $ 1,870.62 | $ 78.72 | $ 711.43 | $ 61.24 | $ 87.01 | $ 63.69 | $ 275.02 | $ 136.77 | $ 223.71 | $ 131.60 |
| **2007** | $ 635.84 | $ 449.99 | $ 1,258.76 | $ 1,101.13 | $ 612.37 | $ 283.46 | $ 111.24 | $ 1,787.79 | $ 88.58 | $ 744.81 | $ 66.41 | $ 96.39 | $ 95.42 | $ 312.70 | $ 172.12 | $ 243.70 | $ 151.84 |
| **2008** | $ 614.09 | $ 508.65 | $ 1,473.88 | $ 1,158.30 | $ 623.32 | $ 353.21 | $ 161.37 | $ 1,712.62 | $ 97.80 | $ 803.70 | $ 76.71 | $ 110.74 | $ 126.24 | $ 349.80 | $ 227.21 | $ 267.02 | $ 168.99 |
| **2009** | $ 706.84 | $ 547.80 | $ 1,472.42 | $ 1,172.00 | $ 689.34 | $ 400.77 | $ 162.15 | $ 1,788.65 | $ 102.82 | $ 801.88 | $ 72.83 | $ 129.33 | $ 121.51 | $ 387.42 | $ 229.51 | $ 282.19 | $ 179.43 |
| **2010** | $ 711.84 | $ 594.01 | $ 1,466.39 | $ 1,131.24 | $ 684.85 | $ 370.85 | $ 157.54 | $ 1,611.61 | $ 103.38 | $ 805.83 | $ 73.62 | $ 136.38 | $ 129.28 | $ 428.51 | $ 248.75 | $ 294.41 | $ 179.99 |
| **2011** | $ 873.86 | $ 568.90 | $ 1,477.00 | $ 1,311.24 | $ 655.31 | $ 377.01 | $ 172.88 | $ 1,809.26 | $ 90.13 | $ 797.25 | $ 68.13 | $ 146.48 | $ 126.82 | $ 324.42 | $ 302.41 | $ 321.50 | $ 192.46 |
| **2012** | $ 992.35 | $ 569.37 | $ 1,479.33 | $ 1,151.00 | $ 646.28 | $ 365.48 | $ 303.65 | $ 2,097.35 | $ 73.27 | $ 1,039.39 | $ 76.29 | $ 165.95 | $ 116.28 | $ 676.57 | $ 346.08 | $ 307.35 | $ 184.49 |
| **2013** | $ 1,070.36 | $ 572.66 | $ 1,578.27 | $ 1,274.27 | $ 607.55 | $ 342.64 | $ 345.24 | $ 2,103.39 | $ 70.03 | $ 1,162.32 | $ 79.89 | $ 161.87 | $ 121.45 | $ 525.02 | $ 351.65 | $ 314.84 | $ 187.43 |
| **2014** | $ 1,148.73 | $ 675.80 | $ 1,612.60 | $ 1,387.32 | $ 569.35 | $ 359.31 | $ 292.33 | $ 2,108.70 | $ 66.53 | $ 1,244.23 | $ 80.03 | $ 177.97 | $ 129.99 | $ 372.14 | $ 362.13 | $ 305.65 | $ 190.27 |
| **2015** | $ 1,194.01 | $ 636.43 | $ 1,711.00 | $ 1,411.00 | $ 645.13 | $ 257.40 | $ 154.47 | $ 2,029.40 | - | $ 1,189.96 | $ 72.03 | $ 156.63 | $ 151.78 | - | $ 291.61 | $ 258.01 | $ 159.76 |

**Appendix B.**

| Country Name | Saudi Arabia | Oman | United Arab Emirates | Kuwait | Lebanon | Jordan | Iraq | Qatar | Syria | Bahrain | Yemen | Egypt | Sudan | Libya | Algeria | Tunisia | Morocco |
| --- | --- | --- | --- | --- | --- | --- | --- | --- | --- | --- | --- | --- | --- | --- | --- | --- | --- |
| 1995 | 71.10502439 | 69.98841 | 73.19227 | 72.90259 | 72.0682 | 70.92573 | 68.51027 | 75.86583 | 71.95676 | 73.53073 | 59.23441 | 66.76068 | 56.70561 | 69.9509 | 68.09837 | 71.57534 | 66.78471 |
| 1996 | 71.44192683 | 70.49059 | 73.45863 | 72.99724 | 72.51717 | 71.09568 | 68.79729 | 76.00144 | 72.20007 | 73.73224 | 59.48571 | 67.2321 | 57.02802 | 70.10102 | 68.49102 | 71.98561 | 67.11817 |
| 1997 | 71.75919512 | 70.97878 | 73.7239 | 73.08639 | 72.98388 | 71.26356 | 69.00678 | 76.13502 | 72.4329 | 73.93429 | 59.72551 | 67.66407 | 57.36449 | 70.22751 | 68.91471 | 72.35268 | 67.45793 |
| 1998 | 72.05421951 | 71.45246 | 73.98751 | 73.169 | 73.46141 | 71.43288 | 69.13856 | 76.26659 | 72.65673 | 74.13437 | 59.95383 | 68.0361 | 57.71056 | 70.3418 | 69.35732 | 72.68354 | 67.8198 |
| 1999 | 72.32390244 | 71.91217 | 74.25002 | 73.2481 | 73.94483 | 71.6051 | 69.19593 | 76.39661 | 72.87363 | 74.33298 | 60.17615 | 68.34368 | 58.06429 | 70.45185 | 69.81429 | 72.98361 | 68.21427 |
| 2000 | 72.56165854 | 72.35937 | 74.50946 | 73.32371 | 74.43171 | 71.77976 | 69.17932 | 76.52159 | 73.10229 | 74.52802 | 60.40444 | 68.58832 | 58.42424 | 70.57254 | 70.28568 | 73.25383 | 68.65215 |
| 2001 | 72.76092683 | 72.79605 | 74.76688 | 73.39585 | 74.92112 | 71.95637 | 69.09 | 76.63851 | 73.36705 | 74.71798 | 60.64922 | 68.78302 | 58.79395 | 70.71678 | 70.77202 | 73.49961 | 69.14029 |
| 2002 | 72.91912195 | 73.2232 | 75.01832 | 73.46405 | 75.4121 | 72.13249 | 68.94202 | 76.74639 | 73.6668 | 74.90078 | 60.91544 | 68.94827 | 59.17693 | 70.88554 | 71.27039 | 73.72139 | 69.67015 |
| 2003 | 73.04068293 | 73.6388 | 75.26383 | 73.52976 | 75.89922 | 72.30461 | 68.75732 | 76.84373 | 73.98178 | 75.0769 | 61.20712 | 69.10407 | 59.57463 | 71.07485 | 71.77432 | 73.92315 | 70.22973 |
| 2004 | 73.13207317 | 74.04141 | 75.50141 | 73.59598 | 76.375 | 72.47376 | 68.56012 | 76.93461 | 74.28002 | 75.24537 | 61.52173 | 69.26041 | 59.98407 | 71.2768 | 72.27583 | 74.10688 | 70.80856 |
| 2005 | 73.2047561 | 74.42654 | 75.72854 | 73.66515 | 76.82898 | 72.6389 | 68.38168 | 77.02454 | 74.48193 | 75.40717 | 61.85529 | 69.42434 | 60.40673 | 71.471 | 72.76141 | 74.27217 | 71.39285 |
| 2006 | 73.27226829 | 74.78824 | 75.9442 | 73.74122 | 77.25015 | 72.80156 | 68.25502 | 77.12015 | 74.4922 | 75.56339 | 62.20083 | 69.59834 | 60.8421 | 71.63107 | 73.21956 | 74.41515 | 71.96785 |
| 2007 | 73.34763415 | 75.12302 | 76.14834 | 73.82515 | 77.63349 | 72.9622 | 68.19654 | 77.22744 | 74.2652 | 75.71456 | 62.54634 | 69.77944 | 61.2871 | 71.73866 | 73.64271 | 74.53541 | 72.52024 |
| 2008 | 73.44139024 | 75.42993 | 76.34041 | 73.91888 | 77.97646 | 73.12229 | 68.21432 | 77.34944 | 73.79846 | 75.86327 | 62.88134 | 69.9631 | 61.7342 | 71.78695 | 74.02532 | 74.63698 | 73.03812 |
| 2009 | 73.55807317 | 75.71144 | 76.52244 | 74.02144 | 78.27756 | 73.28085 | 68.31246 | 77.48763 | 73.12949 | 76.01002 | 63.20032 | 70.15078 | 62.17578 | 71.78051 | 74.36785 | 74.72529 | 73.51346 |
| 2010 | 73.70014634 | 75.97007 | 76.69488 | 74.13285 | 78.53827 | 73.43839 | 68.48217 | 77.64049 | 72.33983 | 76.15532 | 63.49824 | 70.34141 | 62.60076 | 71.73698 | 74.66978 | 74.81127 | 73.94107 |
| 2011 | 73.86360976 | 76.21178 | 76.85978 | 74.25166 | 78.76456 | 73.5939 | 68.70473 | 77.80441 | 71.53944 | 76.29963 | 63.77561 | 70.5349 | 62.99702 | 71.68334 | 74.93756 | 74.90668 | 74.32024 |
| 2012 | 74.03846341 | 76.44202 | 77.01915 | 74.37441 | 78.96541 | 73.7469 | 68.95076 | 77.97237 | 70.85149 | 76.44295 | 64.03339 | 70.7282 | 63.36005 | 71.65007 | 75.1812 | 75.0189 | 74.66066 |
| 2013 | 74.21821951 | 76.66829 | 77.175 | 74.49868 | 79.15032 | 73.89693 | 69.19546 | 78.13973 | 70.37544 | 76.58522 | 64.27707 | 70.92024 | 63.68578 | 71.6591 | 75.41217 | 75.15429 | 74.97015 |
| 2014 | 74.39739024 | 76.89456 | 77.32937 | 74.62349 | 79.32627 | 74.04495 | 69.42561 | 78.30351 | 70.16322 | 76.72546 | 64.50717 | 71.10956 | 63.97473 | 71.71939 | 75.63502 | 75.31434 | 75.25515 |
| 2015 | 74.5744878 | 77.12183 | 77.48424 | 74.74683 | 79.49824 | 74.19102 | 69.63461 | 78.46271 | 70.25266 | 76.8652 | 64.72568 | 71.29566 | 64.22941 | 71.83039 | 75.85529 | 75.49705 | 75.52015 |
